# Thoraco-omphalopagus conjoined twins: comprehensive evaluation with sonography and MRI in first trimester—a rare imaging diagnosis

**DOI:** 10.1093/bjrcr/uaae045

**Published:** 2024-11-23

**Authors:** Harsimran Bhatia, Purnima Aggarwal, Shritik Devkota, Samiksha Lamichhane

**Affiliations:** Department of Radiodiagnosis and Imaging, Post Graduate Institute of Medical Education and Research, Chandigarh 160012, India; Department of Radiodiagnosis and Imaging, Government Medical College and Hospital, Sector 32, Chandigarh, 160030, India; Department of Radiodiagnosis and Imaging, Post Graduate Institute of Medical Education and Research, Chandigarh 160012, India; Department of Radiodiagnosis and Imaging, B.P. Koirala Institute of Health Sciences, Dharan, 56700, Nepal

**Keywords:** first trimester, conjoined twins, siamese twins, thoraco-omphalopagus, monochorionic-monoamniotic, thoracopagus, omphalopagus

## Abstract

Conjoined twins, or Siamese twins as they are commonly called, are a rare and extreme form of monochorionic twinning. Imaging plays an essential role in the diagnosis and follow-up of conjoined twins. While ultrasound is often the screening modality of choice, MRI is carried out for better anatomical delineation and further characterization as and when necessary. We present a unique case of first trimester thoraco-omphalopagus conjoined twins with cystic hygroma who were comprehensively evaluated with sonography and MRI with imaging findings confirmed post-pregnancy termination. The case stresses upon the utility of advanced imaging techniques including foetal MRI that immensely contribute towards a reliable diagnosis.

## Clinical presentation

A 26-year-old primigravida female came to the antenatal clinic at 11^+2^ weeks period of gestation. General physical examination (including blood pressure) was normal. Baseline laboratory (blood and urine) investigations were under normal limits. No significant past medical or surgical history was present. There was no history of twining or congenital defects in the family.

## Imaging findings

**Figure 1.**
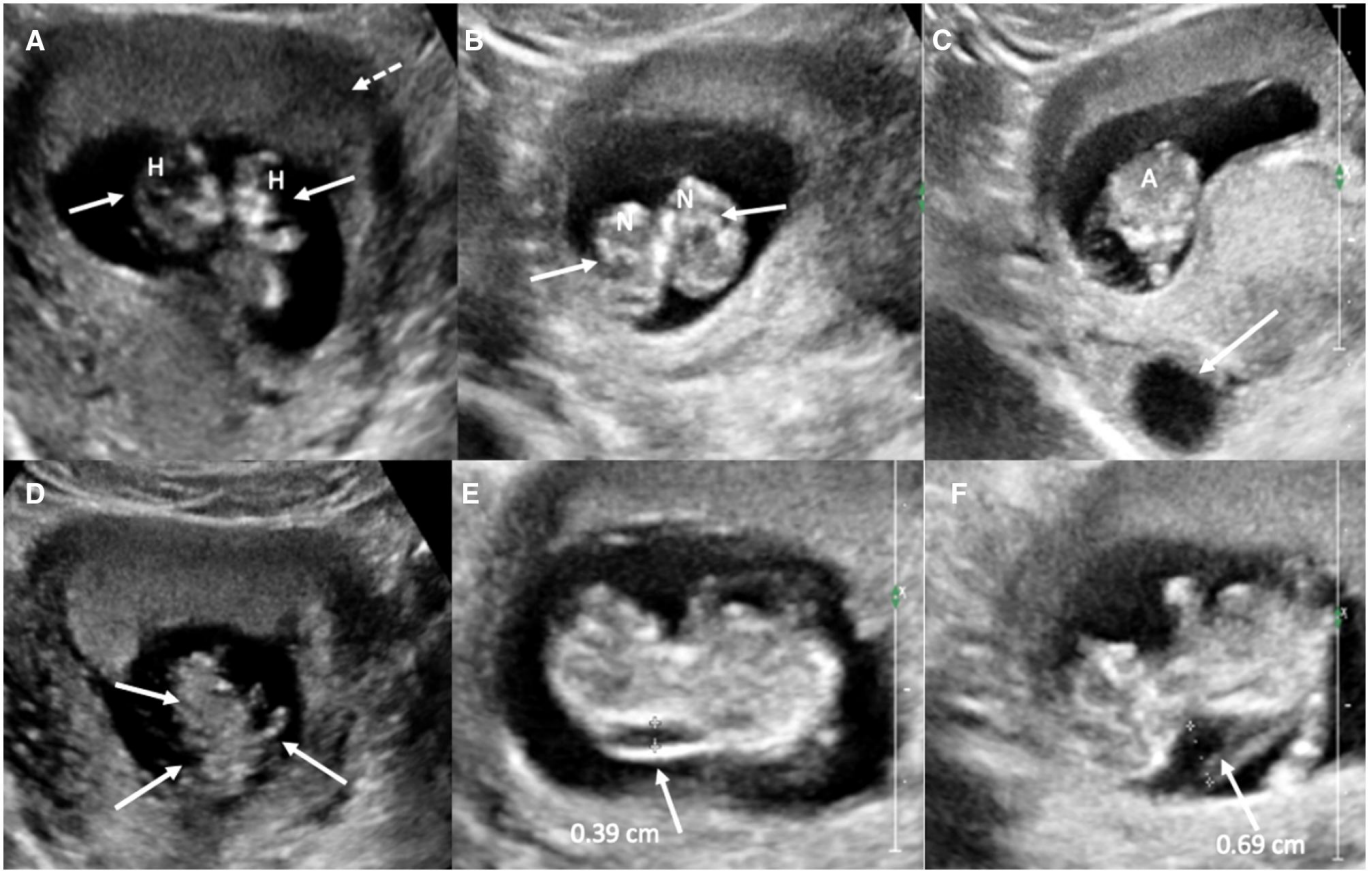
Sonography showed monochorionic monoamniotic twin intrauterine pregnancy. (A and B) Two separate foetal heads and necks (white arrows, denoted by H and N, respectively); (C) single abdominal cavity (denoted by A) and right adnexal cyst (white arrow); (D) multiple limbs (white arrows); and (E and F) increased nuchal translucency (white arrow in E) and cystic hygroma (white arrow in F).

She was referred for a routine transabdominal ultrasonography (USG) that showed a twin pregnancy with foetuses sharing a common amniotic sac and a single placenta. No inter-twin membrane was identified. Both foetuses had separate heads and necks with fusion below the level of the thorax extending till the perineum. There was a single heart, common abdominal cavity with 2 kidneys, 1 liver, and 1 stomach bubble. Thus, a sonographic diagnosis of mono-chorionic mono-amniotic thoraco-omphalopagus conjoined twins was considered. One of the foetuses had increased nuchal translucency (NT) (3.9 mm), while the other had an anechoic cystic collection with echogenic septations along the posterior aspect of neck (maximum thickness of 7 mm) suggestive of cystic hygroma. Exact number of limbs could not be delineated sonographically. In addition, there was a single anteriorly located placenta, single 3-vessel umbilical cord with normal insertion site, adequate liquor, and a simple right adnexal cyst (Figure 1). Crown rump length of the 2 foetuses was 11 weeks 1 day and 10 weeks 4 days, respectively.

The patient further underwent an MRI on 1.5 T (Achieva; Philips Medical Systems) to delineate the exact level of fusion, know the status of the organ sharing, and confirm the sonographic diagnosis of cystic hygroma. BTFE (Balanced Turbo Field Echo) sequence ([T2-weighted gradient echo sequence with following parameters: TR/TE: 4/2 ms; field of view: 210 mm; matrix: 208; pixel size: 1.06 × 1.09 mm; slice thickness: 1.7 mm; and scan time: 3 min]) was conducted without administration of intravenous contrast. It revealed the presence of 2 separate heads and necks with fusion in the region of the thorax and abdomen, single heart, liver, umbilical cord, placenta, increased NT, and cystic hygroma. In addition, 6 limbs were clearly delineated with the foetuses sharing one upper and lower extremity each.

## Differential diagnosis

**Figure 2.**
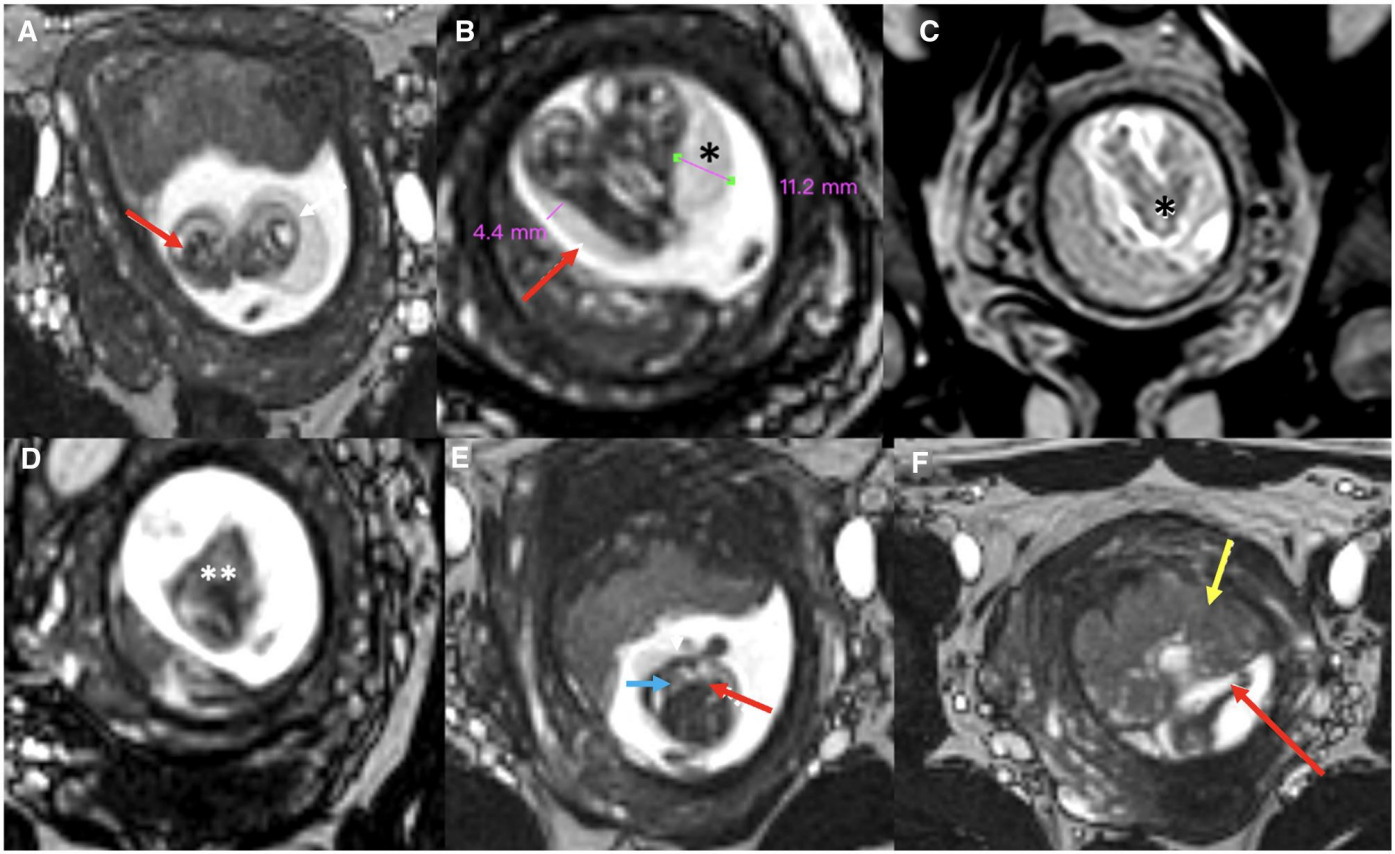
BTFE (Balanced Turbo Field Echo T2-weighted gradient echo sequence) foetal MRI. Sagittal images showed separate foetal heads (red arrows in A) and separate necks (B) with increased NT (nuchal translucency) (red arrow) and cystic hygroma (* in B), with fusion involving thorax (denoted by * in C) and abdomen (denoted by ** in D); (E) coronal image showed single heart (blue arrow) and liver (red arrow); (F) anteriorly located placenta (yellow arrow) and normal insertion site of single umbilical cord (red arrow).

Monochorionic monoamniotic pregnancy was established on USG as well as MRI. The latter further confirmed and characterized fusion and organ sharing. A diagnosis of thoraco-omphalopagus conjoined twins was thus established radiologically (Figure 2).

## Treatment and follow-up

**Figure 3.**
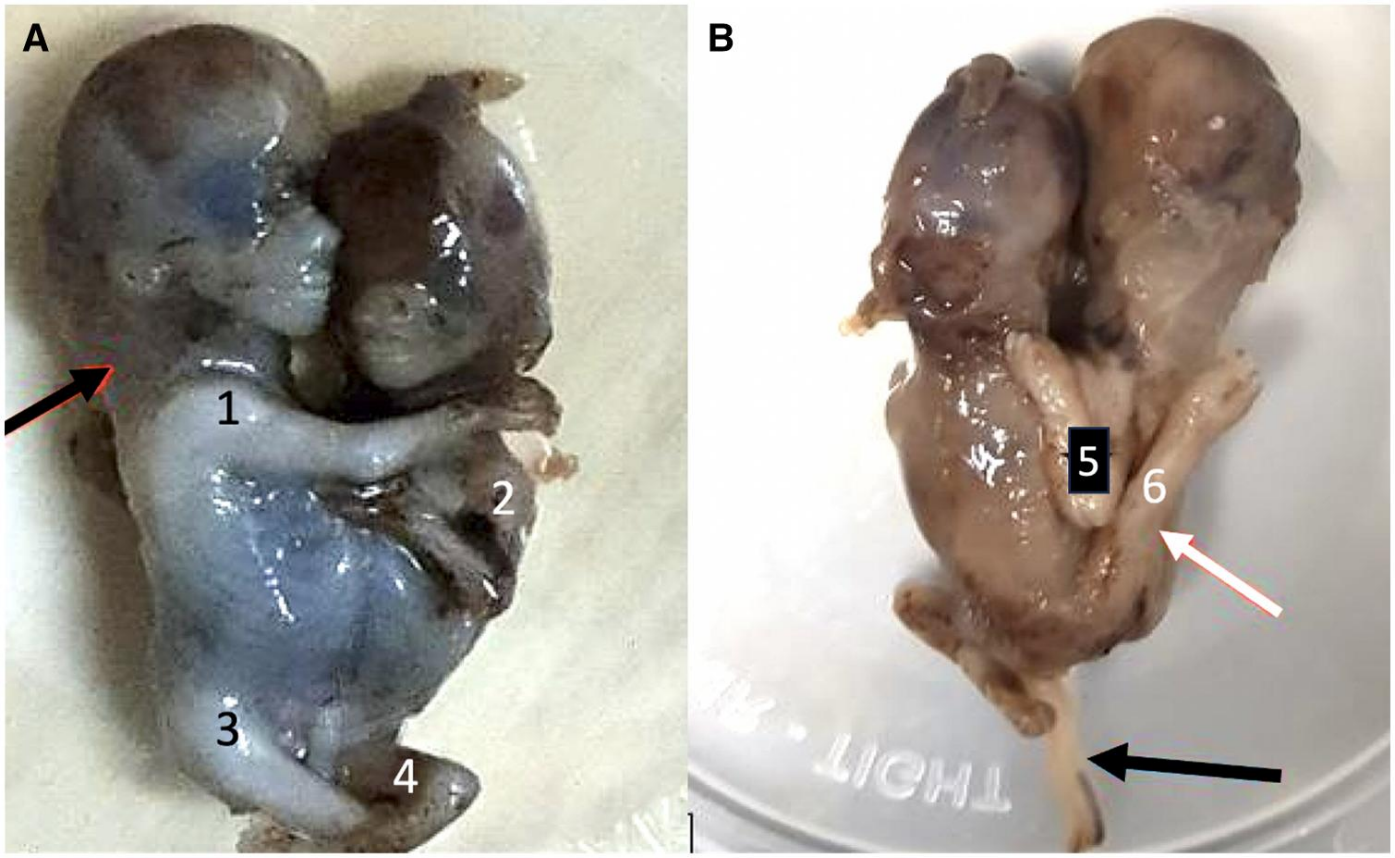
Photographic image of aborted foetuses shows cystic hygroma (black arrow in A) and fusion at thorax-abdomen level; anterior-A and posterior-B views show 4 limbs (numbered 1-4) anteriorly and two limbs (5, 6) posteriorly (one upper and lower) shared by the twins (white arrow in B). Black arrow in B shows single umbilical cord.

Subsequently, she underwent uneventful Medical Termination of Pregnancy after administration of 600 mg of Mifepristone. The imaging findings were confirmed post-pregnancy termination. The aborted foetuses were diagnosed as thoraco-omphalopagus conjoined twins with the presence of cystic hygroma. A single cord and placenta were also appreciated. Patient was then discharged in stable condition the next day (Figure 3).

## Discussion

Conjoined twins are an extreme and rare form of monozygotic twinning with a reported female-to-male ratio of about 3:1.^1^ Since the embryological split occurs after day 13, these twins in addition to sharing the chorion and amnion, also share visceral organs to a variable degree.^2^ Based on the site of fusion, they have been classified as thoracopagus (thorax), omphalopagus (abdomen), pyopagus (sacrum), ischiopagus (pelvis), craniopagus (skull), cephalopagus (face), and rachipagus (back).^3^^,^^4^ Of the various forms, anterior fusion from the mid chest to umbilicus is the most common type. While thoracopagus conjoined twins are fused from the upper thorax to the umbilicus and share a common sternum, diaphragm and upper abdominal wall (with a variable degree of cardiac fusion), omphalopagus twins, on the other hand, are united anteriorly in the umbilical region, having a common liver.^5^^,^^6^

Accurate and early diagnosis with sonography is essential to decide future pregnancy course. Suspicious features that raise a red flag in a first trimester scan of a monochorionic pregnancy include foetuses with heads and body parts at the same level persistently, inseparable body and skin contours, lesser than expected number of limbs, and a variable degree of organ sharing.^7^^,^^8^

Conjoined twins, particularly thoraco-omphalopagus type are associated with other congenital anomalies in about 25% of the cases, ranging from increased NT to congenital heart disease and neural tube defects. While the former anomalies are quite common, cystic hygroma is reportedly a rare congenital malformation in such cases.^9^ Our case is thus unique in demonstrating the common and not-so-common anomalies in these conjoined foetuses.

The diagnosis of anomalous conjoined twins in our case highlights the importance of a first trimester sonographic scan in not only determining the chorionicity and amnionicity accurately, instead it also stresses upon its utility as a tool to search for potential congenital anomalies. Advanced imaging techniques like foetal MRI are being increasingly used in evaluating twin pregnancy, particularly conjoined twins, for better characterization of the site and extent of fusion, to rule out or confirm associated anomalies and to determine the feasibility for surgical separation, as and when necessary. While a thoroughly done sonographic scan in the first trimester is often accurate enough in diagnosing this entity, MRI due to its superior soft tissue contrast and anatomical resolution is required in cases advanced gestational age, maternal obesity, and oligohydramnios where sonography may not suffice in providing complete anatomical details as well as in cases where a diagnosis has been established and surgical separation is contemplated.^10^

## Learning points

- Ultrasound is the screening and primary imaging modality of choice in the evaluation of first trimester twin pregnancy. It not only determines the chorionicity and amnionicity accurately at this stage, in addition, it proves to be an extremely useful tool to rule out congenital malformations.
- The radiologist must be familiar with a number of features that raise a suspicion of rarely encountered monochorionic monoamniotic pregnancy. These include foetuses with heads and body parts at the same level persistently, inseparable body and skin contours, lesser than expected number of limbs, and a variable degree of organ sharing.
- MRI due to its qualities of excellent soft tissue characterization and high spatial resolution contributes immensely in the further evaluation of such conjoined foetuses. Exact level and extent of fusion, degree of organ sharing, and potential congenital anomalies are better characterized to determine feasibility for surgical separation as and when necessary.

## Informed consent

Written informed consent was obtained from the patient(s) for publication of this case review, including accompanying images.
